# Supplementary material for: Comparative efficacy and safety of the artemisinin derivatives compared to quinine for treating severe malaria in children and adults: A systematic update of literature and network meta-analysis
Source: PLoS One. 2022 Jul 20;17(7):e0269391. doi: 10.1371/journal.pone.0269391 (PMC9299386; doi:10.1371/journal.pone.0269391)
Supplement: S1 File — (PDF) [file pone.0269391.s002.pdf]

Supplementary Material

Table A- Example of search terms from previous Cochrane systematic review, updated.

Search set	CIDG SR[a]	CENTRAL	MEDLINE[b]	EMBASE[b]	LILAC[b]	ISI Web of science	Trial Registers[c]
1	malaria	Malaria,ti,ab,MeSH	Malaria,ti,ab MeSH	malaria	malaria	malaria	artemther
2	artemether	Artemether ti, ab	Artemether ti, ab	Artemether ti, ab EMtree	artemether	artemether	severe malaria
3	Artemisinin*	Artemisinin* ti, ab	Artemisini* ti, ab	Artemisinin* ti, ab	Artemisinin*	Artemisinin*	complicated malaria
4	intramuscular	Intramuscular ti, ab	Intramuscular ti, ab	Intramuscular ti, ab	Intramuscular	Intramuscular	artesusnate
5	parenteral	Injection, Intramuscular [MeSH]	Injection, Intramuscular [MeSH]	Intramuscular drug administration [Emtree]	parenteral	parenteral	arteether
6	2 or 3	Parenteral ti,ab	Parenteral ti,ab	Parenteral drug administration [Emtree]	2 or 3	2 or 3	artemisinin
7	4 or 5	2 or 3	2 or 3	2 or 3	4 or 5	4 or 5	
8	1 and 5 and 7	4 or 5 or 6	4 or 5 or 6	4 or 5 or 6	1 and 5 and 7	1 and 5 and 7	
9	-	1 and 7 and 8	1 and 7 and 8	1 and 7 and 8		Randomised clinical trial	
10	-	-				8 and 9	

[a]Cochrane Infectious Diseases Group Specialized Register.
[b]Search terms used in combination with the search strategy for retrieving trials developed by the Cochrane Collaboration.
[c]WHO clinical trial registry platform, ClinicalTrials.gov, and the metaRegister of Controlled Trials (mRCT)

DATA A– Excluded Studies with Reasons

A) The same drugs different doses and combinations

1. Maka DE, Chiabi A, Ndikum V, Achu D, Mah E, Nguefack S, et al. A randomized trial of the efficacy of artesunate and three quinine regimens in the treatment of severe malaria in children at the Ebolowa Regional Hospital, Cameroon. Malar J. 2015;14:429.
2. Krudsood S, Wilairatana P, Vannaphan S, Treeprasertsuk S, Silachamroon U, Phomrattanaprapin W, et al. Clinical experience with intravenous quinine, intramuscular artemether and intravenous artesunate for the treatment of severe malaria in Thailand. Southeast Asian J Trop Med Public Health. 2003;34(1):54-61.

B) Did not measure outcomes of interest

1. Barnes K, Mwenechanya J, Tembo M. Early rectal artesunate is more effective at reducing parasite density compared with intramuscular quinine in people with moderately severe malaria . Evidence-Based Public Heal. 2004:375-376.
2. Byakika-Kibwika P, Achan J, Lamorde M, Karera-Gonahasa C, Kiragga AN, Mayanja-Kizza H, et al. Intravenous artesunate plus Artemisnin based Combination Therapy (ACT) or intravenous quinine plus ACT for treatment of severe malaria in Ugandan children: a randomized controlled clinical trial. BMC Infect Dis. 2017;17(1):794.

C) Placebo or no comparator

1. Gomes M, M.A. F, J.O. G, Warsame M, Agyenyega T, Babiker A, et al. Pre-referral rectal artesunate to prevent death and disability in severe malaria; a placebo-controlled trial. Lancet. 2009.
2. Shukla UK, Damle R, Shukla MM, Singh N. Efiicacy of alpha beta-arteether in children with cerebral malaria. Indian Pediatr. 2001.

D) Not randomized controlled trials

1. Checkley AM, Whitty CJM. Artesunate, artemether or quinine in severe Plasmodium falciparum malaria?. Expert Rev Anti Infect Ther. 2007;5(2):199-204.
2. Woodrow CJ, Planche T, Krishna S. Artesunate versus quinine for severe falciparum malaria. Lancet. 2006;367(9505):110-112.
3. Burch J, Eisenhut M. How do arteether and quinine compare when used to treat severe malaria? Cochrane Clinical Questions;

E) All study participants were initially given clindamycin

1. Nayak KC, Meena R, Kumar S, Gupta BK, Singh VB, Kulkarni V. A Comparative Study of Quinine V / S Artesunate In Severe Malaria Patients In Northwestern Rajasthan, India. Int J Basic Appl Med Sci. 2011;1(1):131-135.

Table B- Frequency distribution of effect modifiers across direct comparisons

	Overall		AMI-QN		AME-QN		ATE-QN		ASU-QN		ASU-AME		ASU-AMI		AMI-AME	
	**n**	**%**	**n**	**%**	**n**	**%**	**n**	**%**	**n**	**%**	**n**	**%**	**n**	**%**	**n**	**%**
**Total comparisons**	41	100	2		20		2		10		3		3		1	
**Publication Year**																
**1989-1999**	24		2	100	11	55	0	0.0	5	50	2	67	3	100	1	100
**2000-2009**	10		0	0.0	6	30	2	100	2	20	0	0.0	0	0.0	0	0.0
**2010-2011**	7		0	0.0	3	15	0	0.0	3	30	1	33	0	0.0	0	0.0
**Age group**																
**Children**	21		1	50	14	70	2	100	3	30	0	0.0	1	33	0	0.0
**Adults**	18		1	50	6	30	0	0.0	5	50	3	100	2	67	1	100
**Both**	2		0	0.0	0	0.0	0	0.0	2	20	0	0.0	0	0.0	0	0.0
**Type of Severe Malaria**																
**Non-specified**	25		1	50	13	65	0	0.0	6	60	2	67	2	67	1	100
**Cerebral malaria only**	16		1	50	7	35	2	100	4	40	1	33	1	33	0	0.0
**Study Continent**																
**Africa**	23		0	0.0	12	60	2	100	3	30	0	0.0	0	0.0	0	0.0
**Asia**	17		2	100	7	35	0	0.0	7	70	3	100	3	100	1	100
**South Pacific**	1		0	0.0	1	5	0	0.0	0	0.0	0	0.0	0	0.0	0	0.0

AMI, Artemisinin; ATE, Arteether; AME, Artemether; ASU, Artesunate; QN, Quinine.
The table shows the number of direct pairwise comparisons.

Fig A-Figure of Assessment of Risk of Bias

Study	Randomisation process	Deviations from the intended interventions	Missing outcome data	Measurement of the outcome	Selection of the reported result
Anh et al 1989	-	+	+	!	+
Win et al 1992	-	+	+	!	+
Karbwang et al 1992	!	+	+	!	+
Hien et al 1992	+	+	+	!	+
Walker et al 1993	+	+	+	!	+
Anh et al 1995	+	+	+	!	+
Karbwang et al 1995	+	+	+	!	+
Hien et al 1996	+	+	+	+	+
Van Hensbroek et al 1996	+	+	+	+	+
Murphy et al 1996	+	+	+	!	+
Vihn et al 1997	!	+	+	!	+
Phuong et al 1997	+	+	+	!	+
Seaton et al 1998	+	+	+	+	+
Taylor et al 1998	+	+	+	!	+
Ojuawo et al 1998	!	+	+	!	+
Olumese et al 1999	!	+	+	!	+
Thuma et al 2000	+	+	+	+	+
Moyou-Somo et al 2001	+	+	+	!	+
Adam et al 2002	!	+	+	+	+
Satti et al 2002	!	+	+	!	+
Newton et al 2003	+	+	+	!	+
Huda et al 2003	!	+	+	+	+
Mohanty et al 2004	-	+	+	!	+
Minta et al 2005	+	+	+	!	+
Dondorp et al 2005	+	+	+	+	+
Haroon et al 2005	!	+	+	!	+
Aguwa et al 2010	-	+	+	!	+
Phu et al 2010	+	+	+	+	+
Dondorp et al 2010	+	+	+	+	+
Eltahir et al 2010	+	+	+	+	+
Osunuga et al 2011	!	+	+	!	+
Abdallah et al 2014	+	+	+	!	+
Bobossi-Serengbe et al 2015	!	+	+	!	+

Low risk +
Some concerns !
High risk -

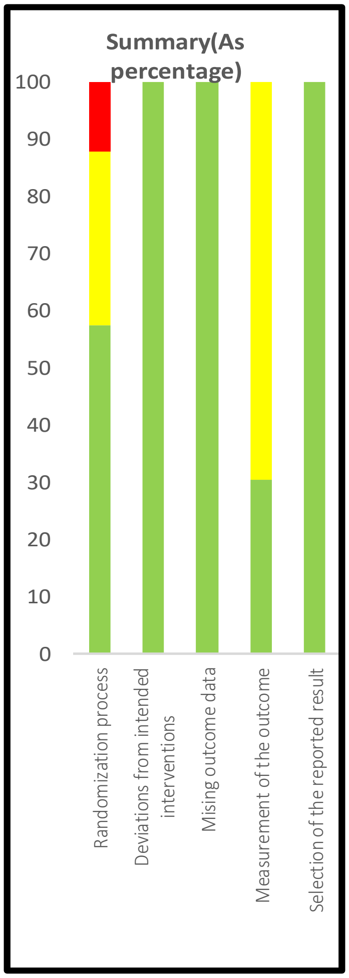

Fig B- Further Investigation of Inconsistency In Coma Recovery Time Adult Analyses Using A. Forest Plot That Compares Direct and Indirect Evidence.

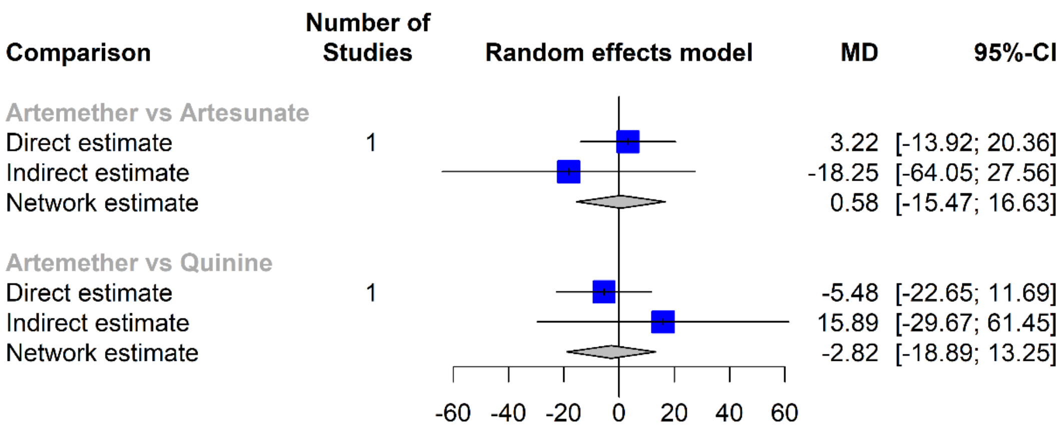

Fig C - Further investigation of inconsistency in Parasite Clearance Time Adult analyses

I. Forest Plot comparing Direct and Indirect Evidence among Adult for Parasite Clearance time

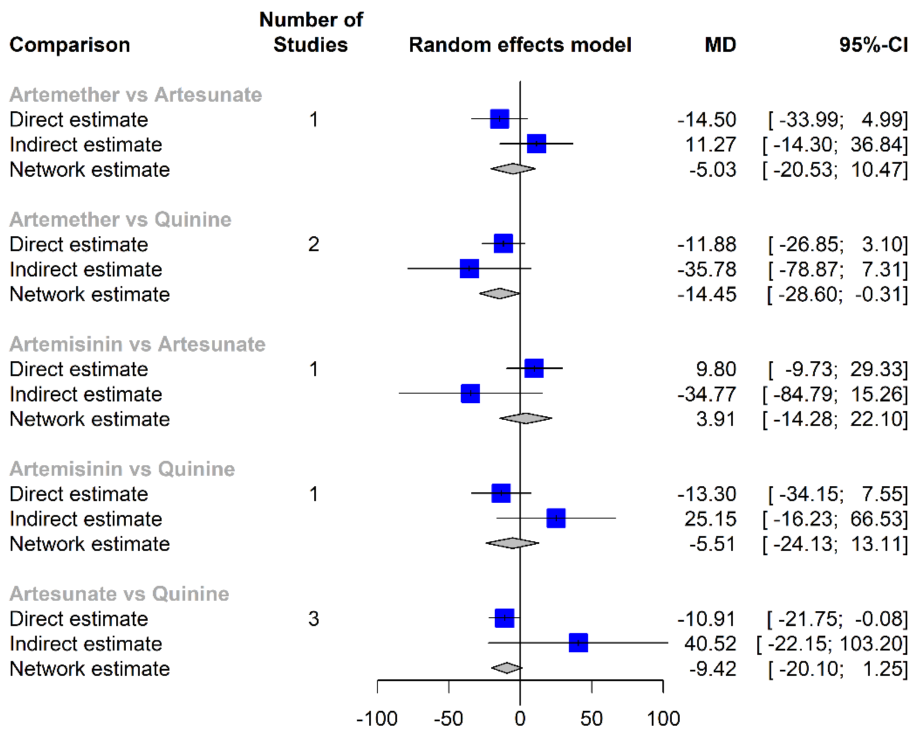

II. Netheat Plot for Parasite Clearance Time for adult Analysis.

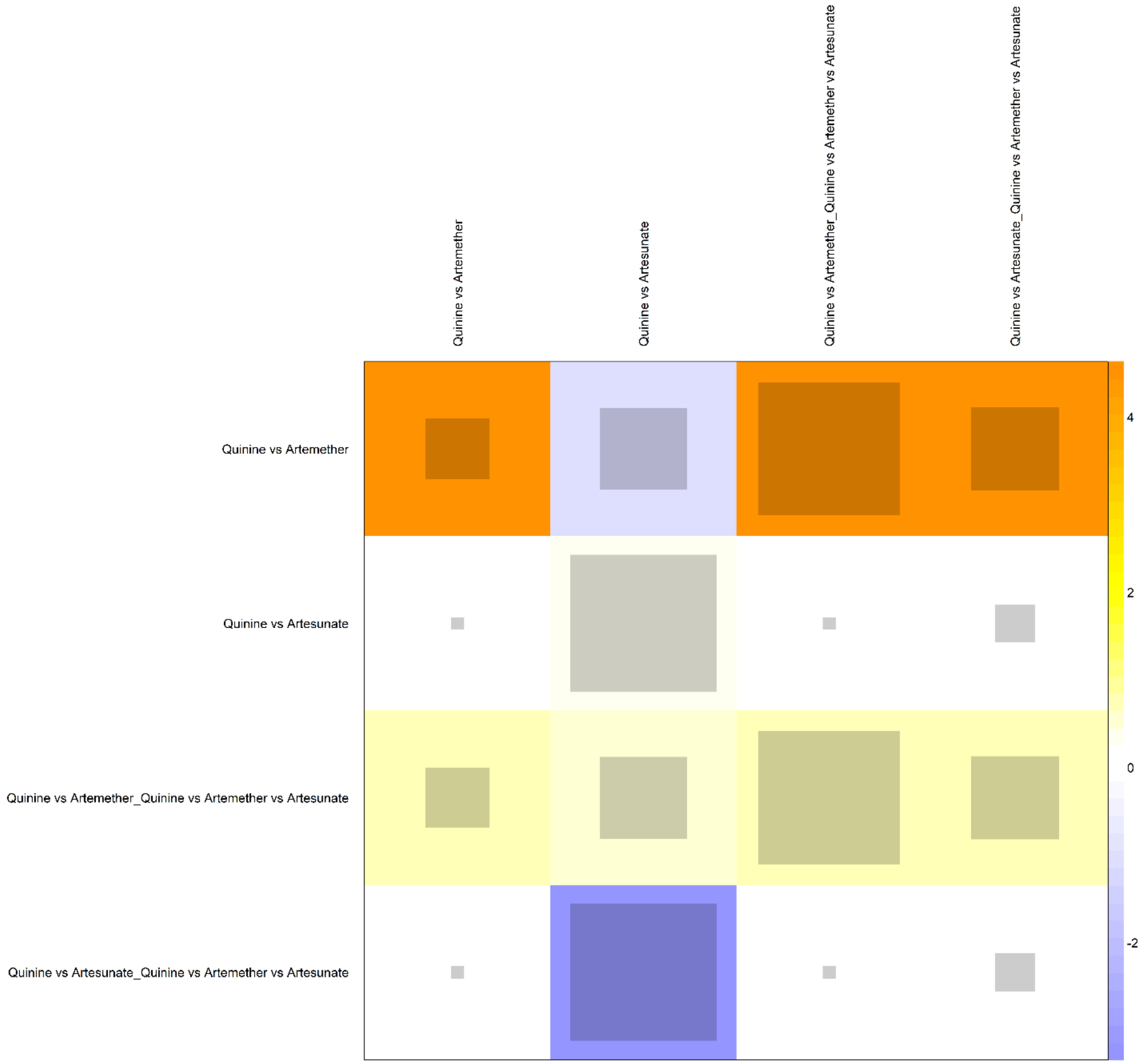

The size of the gray squares is proportional to the contribution of column designs to network estimates of row designs. Warm colors (red) indicate hotspots of high inconsistency.

FIG D- Further Investigation of inconsistency in Fever Clearance Time Adult analyses.

I. Forest Plot comparing Direct and Indirect Evidence among Adult for Fever Clearance time

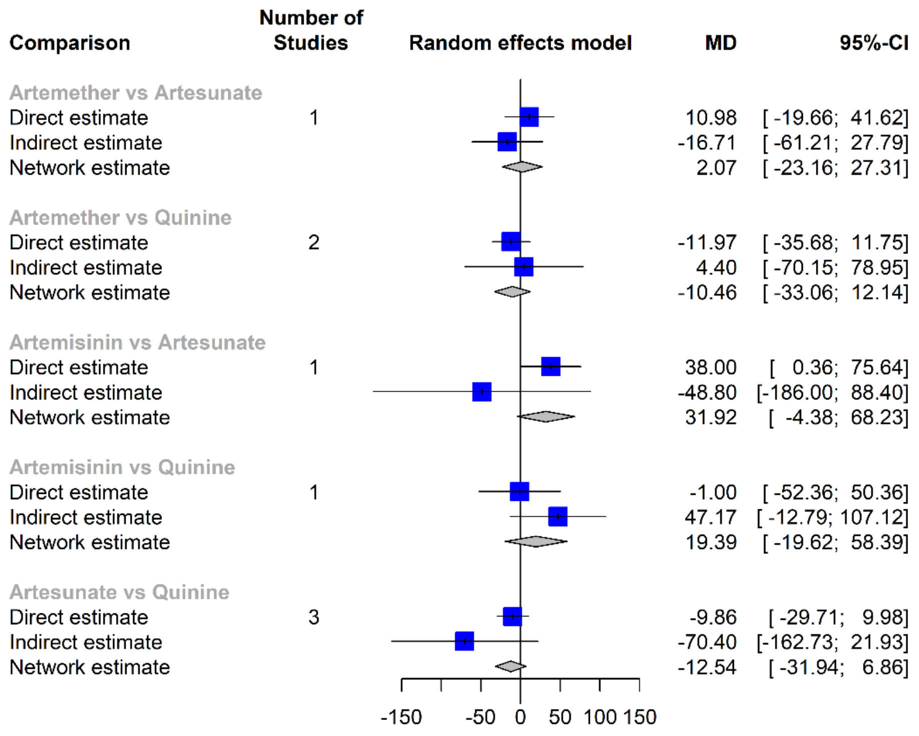

II. Netheat Plot for Fever Clearance Time for Adult Analysis

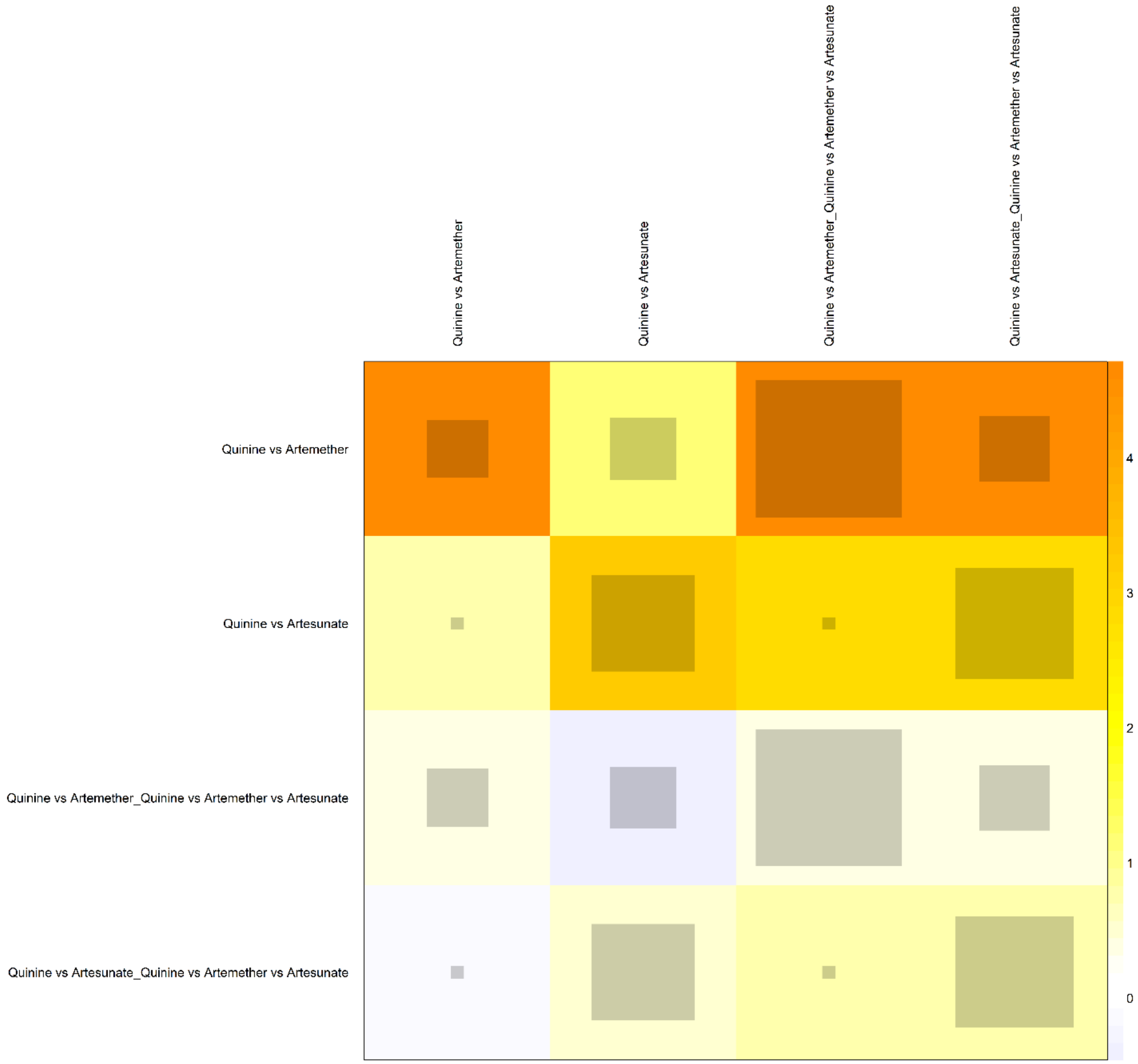

The size of the gray squares is proportional to the contribution of column designs to network estimates of row designs. Warm colors (red) indicate hotspots of high inconsistency.

DATA B- Detailed Discussion of Inconsistency

We anticipated heterogeneity due to a broad research question so we used a random effects model and conducted separate analyses for adults and children to reduce clinical heterogeneity and inconsistency, and increase the clinical relevance of the results. We also used three methods to provide a robust examination of inconsistency and heterogeneity .(2–4) The network results of parasite clearance time were associated with high levels of statistical heterogeneity and inconsistency. This was mostly associated with the evidence coming from the quinine vs artesunate, quinine vs artemether, artemether vs artesunate and the quinine vs artemisinin vs artesunate comparisons. The direct evidence coming from the various quinine vs artesunate and artemether vs quinine studies, were in disagreement with the indirect evidence coming from artemether vs artesunate(5,6) and quinine vs artemisinin vs artesunate(7,8). This is an indication of design inconsistency and may be because indirect evidence from the three-arm trials were mostly from small and earlier RCTs. The various studies were conducted in a wide variety of transmission zones, seasons and different baseline parasite counts, this also accounted for the variability in parasite clearance time analyses. Subgroup analyses and sensitivity analysis could not explain the variability.

We observed that the Q statistics and the netheat plots were mostly in agreement and detected even mild inconsistency, unlike the netsplit forest plots that was able to determine moderate to high inconsistency.

FIG E- Forest Plot of Neurological Sequela Events (results from a pairwise fixed effect meta-analysis model stratified by available drug comparisons using Peto's method for weighting of studies)

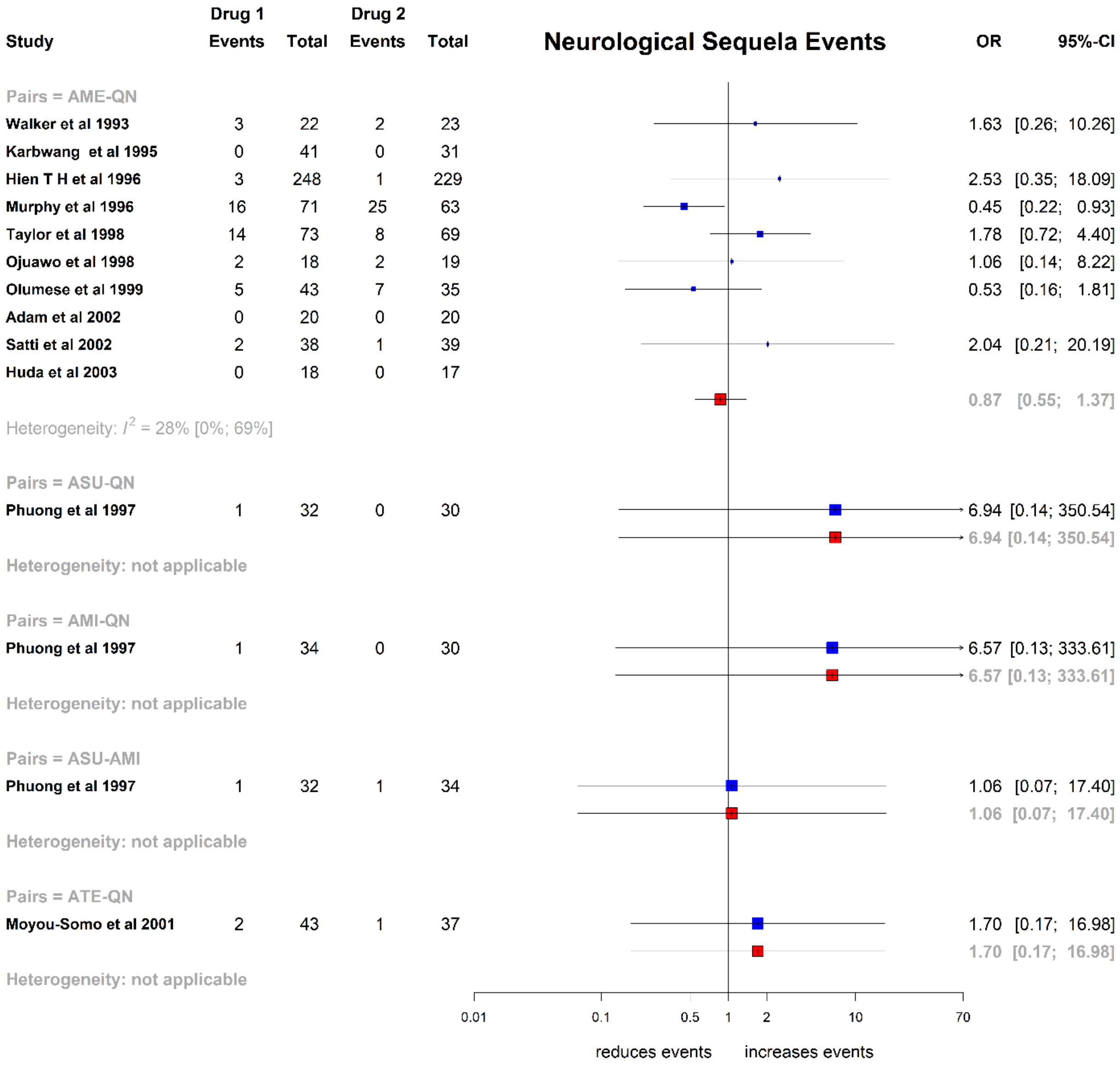

AMI, Artemisinin; ATE, Arteether; AME, Artemether; ASU, Artesunate; QN, Quinine.

FIG F-Network Meta-Analysis effect sizes for all artemisinin derivatives versus quinine for the outcome Hypoglycaemia Events (treatments are ranked by descending probability of being the best at decreasing Hypoglycaemia events)

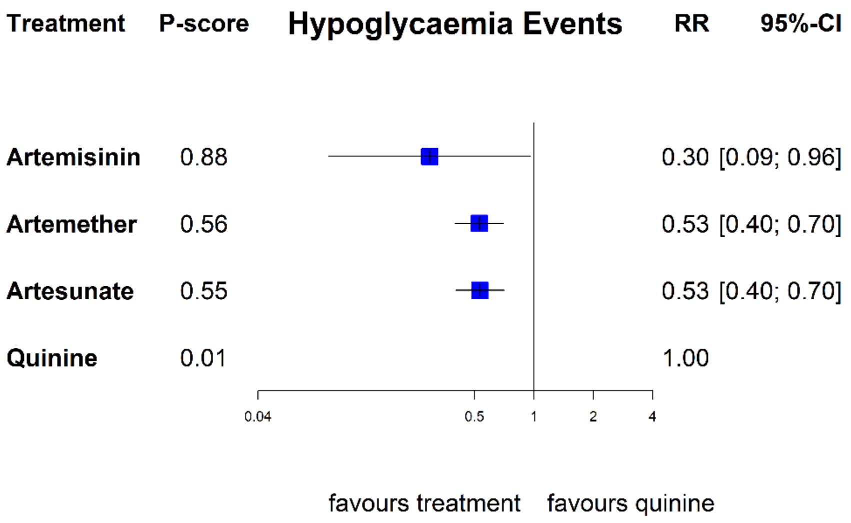

FIG G-Forest Plot of Electrocardiogram Abnormalities (results from a pairwise fixed effect meta-analysis model stratified by available drug comparisons using Peto's method for weighting of studies.

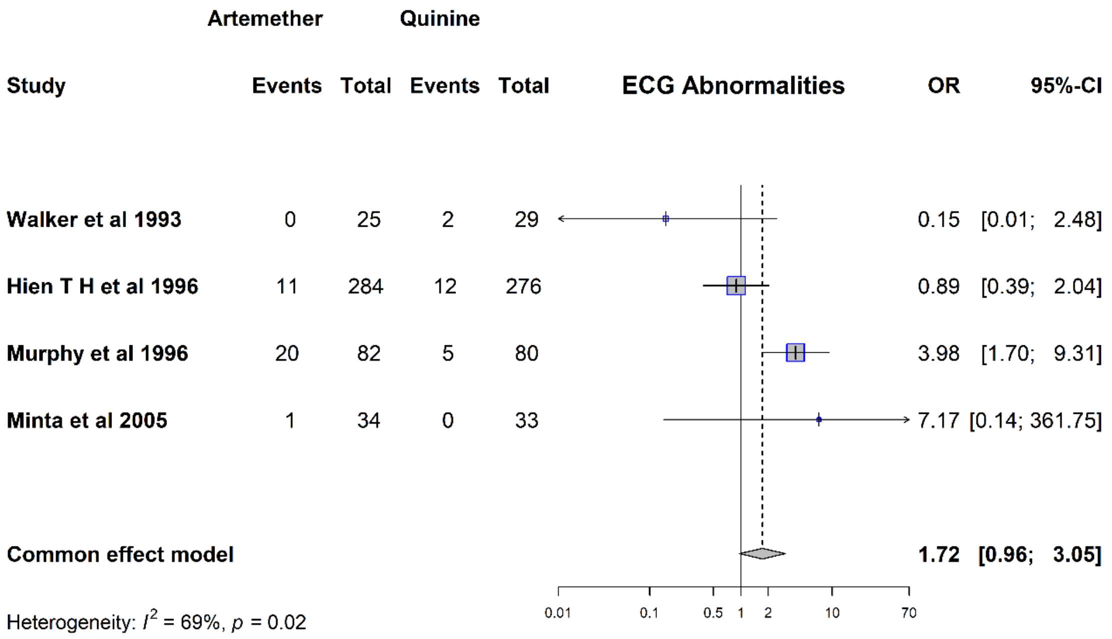

TABLE C- Table of Common Adverse Events reported; number (percentage)

Author Year	Drugs	Hypoglycaemia	Blackwater Fever	Chest Infection	Tinnitus	Urinary Tract Infection	Abscess at injection site	ECG Abnormalities	Any
Walker et al 1993	QN							2(7)	
	AME							0(0)	
Karbwang et al 1995	QN			3(6)		0(0)			
	AME			3(6)		1(2)			
Hien T H et al 1996	QN	69(25)		56(20)		18(7)		12(4)	
	AME	31(11)		64(23)		30(11)		11(4)	
van Hensbroek et al 1996	QN						5(2)		
	AME						1(1)		
Murphy et al 1996	QN							5(6)	
	AME							20(24)	
Phuong et al 1997	QN	9(26)		13(37)		0(0)			
	ASU	6(16)		10(270		2(5)			
	AMI	3(8)		4(11)		1(3)			
Seaton et al 1998	QN	11(79)			5(36)				
	AME	0(0)			0(0)				
Olumese et al 1999	QN	5(10)							
	AME	3(6)							
Thuma et al 2000	QN			8(19)					36(75)
	ATE			7(15)					34(77)
Moyou-Somo et al 2001	QN		1(2)						
	ATE		0(0)						
Adam et al 2002	QN	1(5)							
	AME	0(0)							
Newton et al 2003	QN	15(28)	0(0)						
	ASU	6(10)	1(2)						
Minta et al 2005	QN							1(3)	
	AME							0(0)	
Dondorp et al 2005	QN	19(3)	19(3)						
	ASU	6(1)	49(7)						
Phu et al 2010	ASU	7(4)							
	AME	9(5)							
Dondorp et al 2010	QN	75(3)	18(1)						
	ASU	48(2)	30(1)						
Eltahir et al 2010	QN	1(3)			12(36)				
	ASU	0(0)			0(0)				

AMI, Artemisinin; ATE, Arteether; AME, Artemether; ASU, Artesunate; QN, Quinine

TABLE D- Table of Subgroup Analyses; Network Estimates with global measures of variability [I^2 Statistic (p-value)]

A) Type of Severe Malaria; Mortality RR (95% CI)

Cerebral Malaria Only

AMI		1.72 (0.54; 5.46)		1.04 (0.38; 2.89)
1.30 (0.48; 3.53)	AME	1.68 (0.36; 7.82)		0.85 (0.61; 1.17)
1.54 (0.59; 4.01)	1.19 (0.78; 1.80)	ASU		**0.72 (0.55; 0.93)**
1.47 (0.47; 4.54)	1.13 (0.56; 2.27)	0.95 (0.49; 1.86)	ATE	0.76 (0.41; 1.40)
1.11 (0.43; 2.86)	0.86 (0.62; 1.18)	**0.72 (0.55; 0.94)**	0.76 (0.41; 1.40)	QN
Total variability	0% (p=0.14)			
Heterogeneity	11% (p=0.33)			
Inconsistency	57% (p=0.07)			

Not Specified

AMI	1.59 (0.57 to 4.39)	1.50 (0.72; 3.14)	0.76 (0.22; 2.59)
1.22 (0.63; 2.37)	AME	1.50 (0.87; 2.58)	**0.71 (0.54; 0.92)**
1.53 (0.81; 2.92)	1.26 (0.93; 1.70)	ASU	**0.61 (0.48; 0.77)**
0.90 (0.47; 1.72)	**0.74 (0.58; 0.94)**	**0.58 (0.47; 0.73)**	QN
Total variability	0% (p=0.76)		
Heterogeneity	0% (p=0.69)		
Inconsistency	0% (p=0.65)		

B) Study Continent; Mortality RR (95% CI)

Africa

AME			0.96 (0.76; 1.21)
1.20 (0.90; 1.61)	ASU		0.80 (0.67; 0.95)
1.27	1.06	ATE	0.76

Asia

AMI	1.59 (0.56; 4.47)	1.57 (0.84; 2.93)	0.92 (0.43; 1.98)
1.44 (0.81; 2.56)	AME	1.51 (0.89; 2.56)	0.59 (0.43; 0.80)
1.55	1.08	ASU	0.64

(0.69; 2.32)	(0.59; 1.89)		(0.43; 1.32)
0.96<br>(0.76; 1.21)	0.80<br>(0.67; 0.95)	0.76<br>(0.43; 1.32)	QN
Total variability	0.0% (p=0.86)		
Heterogeneity	0.0% (p=0.86)		
Inconsistency	NA (p=NA)		

(0.90; 2.65)	(0.78; 1.48)		(0.51; 0.81)
0.94<br>(0.55; 1.62)	0.66<br>(0.50; 0.86)	0.61<br>(0.49; 0.76)	QN
Total variability	5% (p=0.40)		
Heterogeneity	0% (p=0.51)		
Inconsistency	30% (p=0.26)		

C) Neurological Sequela Events; RR (95% CI)

Acute Neurological Sequela

AME			0.74<br>(0.57 to 0.97)
0.55<br>(0.36 to 0.82)	ASU		1.36<br>(1.01 to 1.83)
0.69<br>(0.36; 1.32)	1.26<br>(0.65 to 2.44)	ATE	1.08<br>(0.60 to 1.96)
0.74<br>(0.57 to 0.97)	1.36<br>(1.01 to 1.83)	1.08<br>(0.60 to 1.96)	QN
Total variability	0% (p=0.50)		
Heterogeneity	0% (p=0.50)		
Inconsistency	NA (p=NA)		

Persistent Neurological Sequela

AME			0.72<br>(0.29 to 1.74)
0.66<br>(0.23 to 1.88)	ASU		1.09<br>(0.62 to 1.91)
0.61<br>(0.12 to 3.02)	0.93<br>(0.22 to 3.93)	ATE	1.18<br>(0.31 to 4.46)
0.72<br>(0.29 to 1.74)	1.09<br>(0.62 to 1.91)	1.18<br>(0.31 to 4.46)	QN
Total variability	0% (p=0.38)		
Heterogeneity	0% (p=0.38)		
Inconsistency	NA (p=NA)		

AMI, Artemisinin; ATE, Arteether; AME, Artemether; ASU, Artesunate; QN, Quinine.
This table is read from left to right; the treatment in the left vs the treatment to the right of estimates.
The upper part of the diagonal presents results of direct evidence.
The lower diagonal (in grey) provides summary of network estimates (both direct and indirect results) with variability measures at the bottom

FIG H- Forest Plots comparing reported means and standard deviations only with analyses that included conversions with Wang's method treatments against quinine

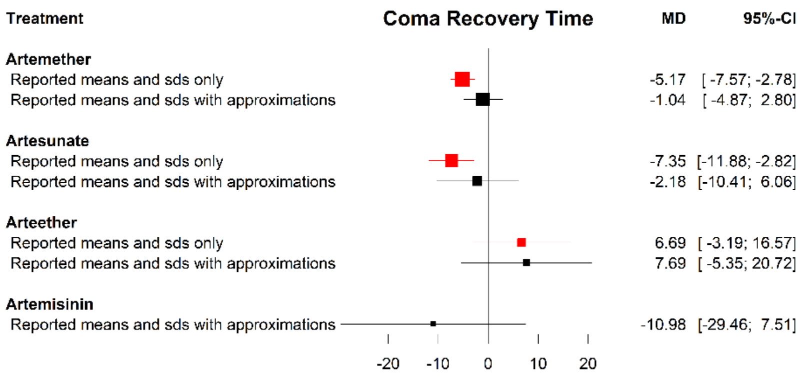

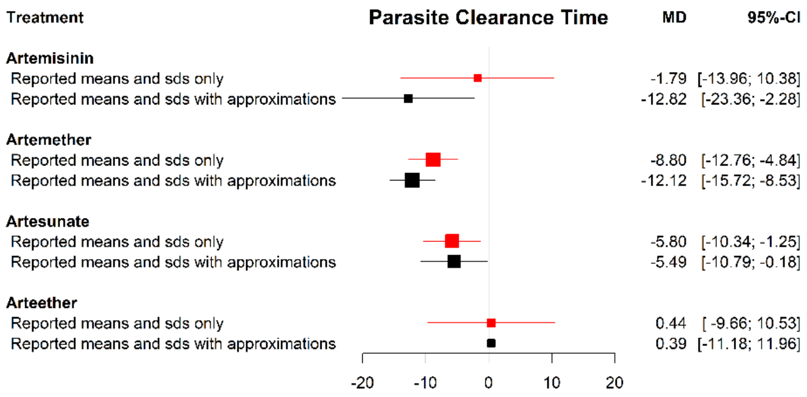

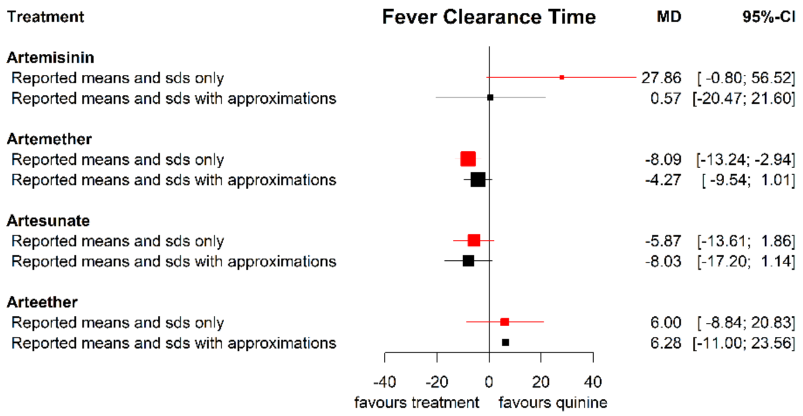

TABLE E- League table of NMA results with measures of variability [I^2 Statistic, p-value] for age groups combined

A) Mortality; RR (95% CI)

n=34 and N=11349

ASU	0.66<br>(0.39 to 1.10)	0.64<br>(0.35 to 1.18)		0.74<br>(0.65 to 0.83)
0.88<br>(0.72 to 1.08)	AME	0.63<br>(0.23 to 1.74)		0.79<br>(0.66 to 0.95)
0.68<br>(0.41 to 1.15)	0.77<br>(0.45 to 1.33)	AMI		0.92<br>(0.44 to 1.96)
0.96<br>(0.54 to 1.70)	1.09<br>(0.61 to 1.95)	1.40<br>(0.65 to 3.01)	ATE	0.76<br>(0.43 to 1.32)
0.73<br>(0.64 to 0.82)	0.82<br>(0.69 to 0.98)	1.06<br>(0.63 to 1.78)	0.76<br>(0.43 to 1.32)	QN
Total variability	0% (p=0.57)		n	34
Heterogeneity	0% (p=0.71)		N	11349
Inconsistency	29% (p=0.20)			

B) Coma Recovery Time; MD, hours. (95% CI)

n=22 and N=3548

ASU		-30.42<br>(-62.15 to 1.32)		-3.36<br>(-12.32 to 5.60)
-1.14<br>(-9.69 to 7.41)	AME	3.42<br>(-8.38 to 15.21)		-1.37<br>(-5.23 to 2.50)
8.80<br>(-10.64 to 28.24)	9.94<br>(-8.88 to 28.76)	AMI		-2.41<br>(-22.65 to 17.82)
-9.86<br>(-25.28 to 5.56)	-8.72<br>(-22.31 to 4.87)	-18.66<br>(-41.28 to 3.96)	ATE	7.69<br>(-5.35 to 20.72)
-2.18<br>(-10.41 to 6.06)	-1.04<br>(-4.87 to 2.80)	-10.98<br>(-29.46 to 7.51)	7.69<br>(-5.35 to 20.72)	QN

Total variability	55% (p=0.001)		n	22
Heterogeneity	57.6% (p=0.001)		N	2334
Inconsistency	39% (p=0.16)			

C) Parasite Clearance Time; MD, hours. (95% CI)

n=24 and N=3517

ASU	23.18<br>(12.44 to 33.92)	-2.05<br>(-12.81 to 8.71)		-9.95<br>(-15.65 to -4.24)
6.64<br>(0.64 to 12.64)	AME			-10.50<br>(-14.19 to -6.80)
7.33<br>(-2.74 to 17.40)	0.70<br>(-10.29 to 11.68)	AMI		-29.36<br>(-42.61 to -16.10)
-5.88<br>(-18.60 to 6.85)	-12.51<br>(-24.63 to -0.40)	-13.21<br>(-28.86 to 2.44)	ATE	0.39<br>(-11.18 to 11.96)
-5.49<br>(-10.79 to -0.18)	-12.12<br>(-15.72 to -8.53)	-12.82<br>(-23.36 to -2.28)	0.39<br>(-11.18 to 11.96)	QN
Total variability	79.4% (p<0.001)		n	24
Heterogeneity	61.5% (p<0.001)		N	3517
Inconsistency	93.6% (p<0.001)			

D) Fever Clearance Time; MD (95% CI)

n=25 and N=3548

ASU	-19.37<br>(-38.79 to 0.05)	-10.52<br>(-34.76 to 13.72)		-3.52<br>(-13.24 to 6.20)
-3.77<br>(-13.91 to 6.38)	AME			-5.22<br>(-10.57 to 0.12)
-8.60<br>(-29.39 to 12.19)	-4.83<br>(-26.40 to 16.73)	AMI		-6.93<br>(-32.62 to 18.77)
-14.31<br>(-33.87 to 5.25)	-10.55<br>(-28.61 to 7.52)	-5.71<br>(-32.93 to 21.51)	ATE	6.28<br>(-11.00 to 23.56)
-8.03<br>(-17.20 to 1.14)	-4.27<br>(-9.54 to 1.01)	0.57<br>(-20.47 to 21.60)	6.28<br>(-11.00 to 23.56)	QN

Total variability	82.0% (p<0.001)	n	25
Heterogeneity	61.5% (p<0.001)	N	3548
Inconsistency	15.6% (p=0.004)		

RR, Risk Ratio; MD, Mean Difference; AMI, Artemisinin; ATE, Arteether ; AME, Artemether; ASU, Artesunate; QN, Quinine; p, p-values; n, number of RCTs; N, number of participants

This table is read from left to right; the treatment in the left versus the treatment to the right of estimates.

The upper part of the diagonal presents results of direct evidence.

The lower diagonal (in grey) provides summary of network estimates (both direct and indirect results) with variability measures at the bottom

FIG I. Network graph of treatment comparisons for mortality among age groups combined

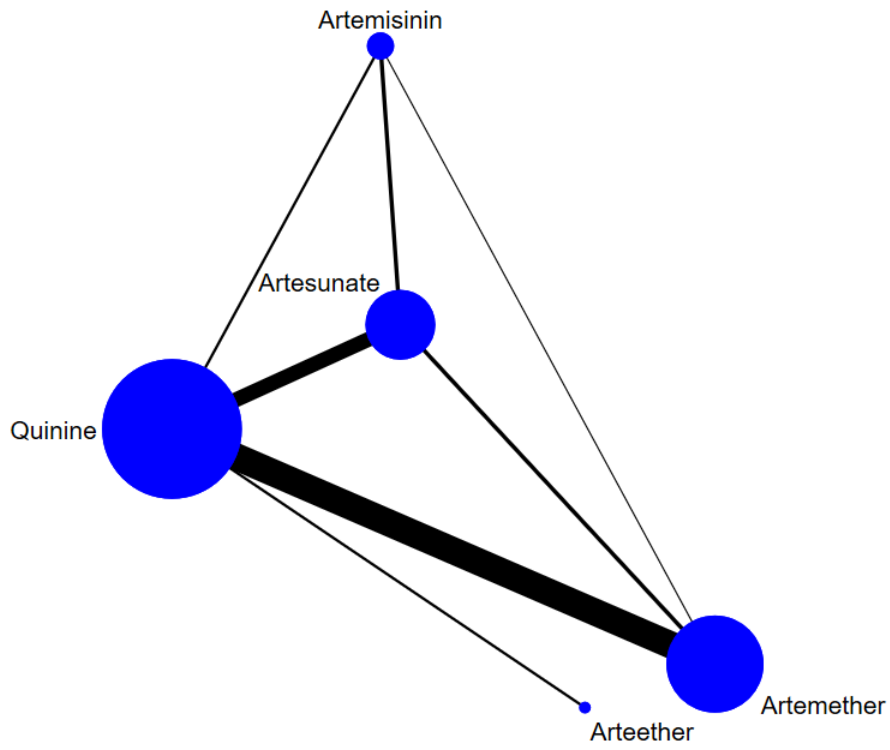

The blue nodes are proportional to the number of participants allocated to that drug. The thickness of the black edges is proportional to the number of studies comparing the drugs on each side of the edge.

DATA C- Grading of evidence using CINeMA based on mortality

The quality of evidence assessment was based on the primary outcome. Although a lot of efforts were made by the previous Cochrane systematic reviews, Eggers test and funnel plot suggested presence of publication bias. We downgraded all comparisons to suspected for reporting bias for both children and adult analyses.

The within-study bias for comparisons as illustrated below was based on the assessment of risk of bias per RCT. Indirectness was downgraded to high for studies involving arteether and artemisinin since they were few and not fairly distributed across potential effect modifiers. The studies involving artemether and artesunate remained low since they were sufficiently distributed across effect modifiers. Imprecision, heterogeneity and incoherence scores were all generated by the CINeMA webpage and consistent with our judgement.

The overall judgement for all the comparisons in all analyses are presented in the table below. The quality of evidence generated for artesunate vs quinine was moderate among children and low among adults. All the other comparisons among children and adults were of very low quality.

Table F. Grading of evidence using CINeMA based on mortality

Comparison	Within-study bias	Reporting bias	Indirectness	Imprecision	Heterogeneity	Incoherence	Confidence rating
**Children**							
**Mixed Evidence**							
Arteether vs Quinine	Some concerns	Some concerns	Major concerns	Major concerns	No concerns	No concerns	Very Low
Artemether vs Quinine	Some concerns	Some concerns	No concerns	Major concerns	No concerns	No concerns	Very Low
Artemisinin vs Artesunate	Some concerns	Some concerns	Some concerns	Major concerns	No concerns	No concerns	Very Low
Artemisinin vs Quinine	Some concerns	Some concerns	Major concerns	Major concerns	No concerns	No concerns	Very Low
Artesunate vs Quinine	No concerns	Some concerns	No concerns	No concerns	No concerns	No concerns	Moderate
**Indirect Evidence**							
Arteether vs Artemether	Some concerns	Some concerns	Some concerns	Major concerns	No concerns	No concerns	Very Low
Arteether vs Artemisinin	Some concerns	Some concerns	Major concerns	Major concerns	No concerns	No concerns	Very Low
Arteether vs Artesunate	No concerns	Some concerns	Some concerns	Major concerns	No concerns	No concerns	Very Low
Artemether vs Artemisinin	Some concerns	Some concerns	Some concerns	Major concerns	No concerns	No concerns	Very Low
Artemether vs Artesunate	Some concerns	Some concerns	No concerns	Major concerns	No concerns	No concerns	Very Low
**Adults**							
**Mixed Evidence**							
Artemether vs Artemisinin	Some concerns	Some concerns	Some concerns	Major concerns	No concerns	No concerns	Very Low
Artemether vs Artesunate	Some concerns	Some concerns	No concerns	Major concerns	No concerns	No concerns	Very Low
Artemether vs Quinine	Some concerns	Some concerns	No concerns	No concerns	Some concerns	No concerns	Low
Artemisinin vs Artesunate	Some concerns	Some concerns	Major concerns	Major concerns	No concerns	No concerns	Very Low
Artemisinin vs Quinine	Some concerns	Some concerns	Some concerns	Major concerns	No concerns	No concerns	Very Low
Artesunate vs Quinine	No concerns	Some concerns	No concerns	No concerns	No concerns	No concerns	Moderate
