# Supplementary material for: Comparative efficacy and safety of the artemisinin derivatives compared to quinine for treating severe malaria in children and adults: A systematic update of literature and network meta-analysis
Source: PLoS One. 2022 Jul 20;17(7):e0269391. doi: 10.1371/journal.pone.0269391 (PMC9299386; doi:10.1371/journal.pone.0269391)
Supplement: Attachment — Submitted filename: Response to Reviewers.pdf [file pone.0269391.s003.pdf]

REBUTTAL LETTER

We thank the editor and the two reviewers for their comments on our manuscript and suggestions for improvement, which have thoroughly incorporated into this new and improved revised version of our manuscript. Below is our response to each point raised by the academic editor and reviewers. We hope that we satisfyingly addressed them and that the manuscript will be now suited for publication.

Sincerely,

On behalf of all authors

Nicholas Nyaaba (RGN, MSc)

ACADEMIC EDITOR

Journal Requirements:

When submitting your revision, we need you to address these additional requirements.

1. Please ensure that your manuscript meets PLOS ONE's style requirements, including those for file naming. The PLOS ONE style templates can be found at

https://journals.plos.org/plosone/s/file?id=wjVg/PLOSOne_formatting_sample_main_body.pdf

Response: Supplementary materials were edited to include 'Fig' or 'Table' as appropriate.

https://journals.plos.org/plosone/s/file?id=ba62/PLOSOne_formatting_sample_title_authors_affiliations.pdf

Response: Corresponding author's name was added, and initials were also included after the corresponding author's email. The second group of contributors was also edited to "&JMOM and JH are Joint Senior Authors".

2. Thank you for stating the following in the Acknowledgments Section of your manuscript:

"Initial part of this review was done as part of Nicholas Nyaaba's MSc from the University of Oxford which was supported by the Ghana Education Trust Fund (GETFund)."

We note that you have provided additional information within the Acknowledgements Section that is not currently declared in your Funding Statement. Please note that funding information should not appear in the Acknowledgments section or other areas of your manuscript. We will only publish funding information present in the Funding Statement section of the online submission form.

Please remove any funding-related text from the manuscript and let us know how you would like to update your Funding Statement. Currently, your Funding Statement reads as follows:

"The authors received no funding for this work."

Please include your amended statements within your cover letter; we will change the online submission form on your behalf.

Response: We have removed funding information from the Acknowledgement and Funding Statement has been amended as

"This study did not directly receive any funding. Initial part of this review was done as part of Nicholas Nyaaba's MSc from the University of Oxford which was supported by the Ghana Education Trust Fund (GETFund) and the Ghana Armed Forces Medical Services (GAFMS). The funders had no role in study design, data collection and analysis, decision to publish, or preparation of the manuscript."

This is included in the Cover Letter.

3. PLOS requires an ORCID iD for the corresponding author in Editorial Manager on papers submitted after December 6th, 2016. Please ensure that you have an ORCID iD and that it is validated in Editorial Manager. To do this, go to 'Update my Information' (in the upper left-hand corner of the main menu), and click on the Fetch/Validate link next to the ORCID field. This will take you to the ORCID site and allow you to create a new iD or authenticate a pre-existing iD in Editorial Manager. Please see the following video for instructions on linking an ORCID iD to your Editorial Manager account: https://www.youtube.com/watch?v=_xcclfuvtxQ

Response: ORCID iD for corresponding author has been created and information has been updated.

Reviewer #1:

This systematic review and network meta-analysis aimed to assess the efficacy and safety of the artemisinin derivatives and quinine for treating severe P. falciparum malaria in children and adults by updating previous published Cochrane reviews. I have focused my review on the methodological and statistical aspects of this manuscript.

Response: Thank you very much for the time dedicated to reviewing our manuscript. We acknowledge that the Reviewer raises very important concerns, most of which are as a result of our efforts to manage the word count. We subsequently, amended the manuscript to resolve these concerns.

Major comments:

1. The statistical methods section of this review is lacking many details that are either required for a network meta-analysis or are needed to align with the results presented by the authors. I strongly suggest that the authors consult the paper by Chaimani et al 2017, which provides more details than the PRISMA NMA for conducting a network meta-analysis. The authors should also be aware that the PRISMA guidelines have been updated and PRISMA 2020 (Page 2021) should be used in conjunction with PRISMA NMA.

Chamaini et al. Additional considerations are required when preparing a protocol for a systematic review with multiple interventions. Journal of Clinical Epidemiology. 2017: 83; 65-74.

Page et al. The PRISMA 2020 statement: an updated guideline for reporting systematic reviews. BMJ. 2021; 372:n71.

Response: We have added more subsections with more details to address this concern. In our attempt to manage the word count, we missed many of these details. We have also updated our reference (see reference number 21) and provided a more useful PRISMA table to accompany this manuscript re-submission.

2. The authors have converted medians (range/inter-quartile range) to mean (Standard deviation) and used this in the main analysis. Median and (range/inter-quartile range)

are reported when the data do not have a normal distribution. By converting these data to mean (Standard deviation) the authors are assuming a normal distribution. Converting summary statistics for skewed data to summary staitstics used for normally distributed data doesn't make sense. The authors should produce a meta-analysis of ratios of geometric means when skewed data are reported on the raw scale. Further details of methods used to produce a meta-analysis of skewed data are available in Higgins et al 2008.
Higgins, White and Anzures-Cabrera. Meta-analysis of skewed data: combining results reported on log-transformed or raw scales. Statistics in Medicine. 2008; 27;6072-92.

Response: Thank you. Please, notice that the methods we applied are those recommended in the Cochrane Handbook of Systematic Reviews. Specifically, the methods by Wan et al has been recommended because it incorporates sample size compared to older methods (Hozo et al and Blands). Cognizant of the limitations mentioned, we considered excluding these studies in which the median was reported, and we did not find them to be very influential on the main results. Furthermore, our main outcome was not a continuous variable. We therefore believe this is unlikely to have major influence on the results and conclusions of our study. We also believe that it was an opportunity to review this method on real live data.

Hozo SP, Djulbegovic B, Hozo I: Estimating the mean and variance fromthemedian, range, and the size of a sample. BMCMed Res Methodol 2005, 5:13.

Bland M: Estimating mean and standard deviation from the sample size, three quartiles, minimum, and maximum. International Journal of Statistics in Medical Research, in press. 2014.

3. The methods section would benefit from more subsection, including a section that explicitly states that inclusion/exclusion criteria for the study.

Response: Thank you. We have now added an inclusion and exclusion subsection in the Methods (see lines 104-112)

4. The eligibility criteria for a network meta-analysis should clearly consider the transitivity assumption. The transitivity assumption is the fundamental assumption underlying the validity of a network meta-analysis. The authors have not considered the transitivity assumption for their network meta-analysis, which is a fundamental error when undertaking a network meta-analysis.

Response: Thank you. We assessed transitivity at two levels to show the distribution of studies across effect modifiers; Table 2 at the study level and S2 Table at the comparison level. We have added a subsection in the Methods/Results? to clearly address this concern (lines 138-142). We also provided explanations in the Result session (lines 208-211).

"Sixteen out of 17 RCTs from Africa were conducted among children and 11 out of 12 RCTs conducted among adults were in Asia. Out of the 12 cerebral malaria only RCTs, eight were from Africa and four from Asia."

In the Discussion (lines 414-418) we also stated that

"We detected an unequal distribution of RCTs in age groups among Asian and African population; sixteen out of 17 RCTs from Africa were conducted among children and 11 out of 12 RCTs conducted among adults were in Asia. The earliest systematic reviews found that artemether was more effective among Asian adults than in African children [13,18]. This has partly motivated subsequent RCTs along these subgroups"

5. The statistical methods section needs to provide an explanation of how the network diagram is created (i.e. what do the size of the nodes and thickness of the edges represent).

Response: Thank you. We have added a description in the Methods Section of the how the network diagrams were created (see lines 162-165).

6. The authors conduct a meta-analysis of rare events (i.e. analysis of serious adverse events and neurological sequalae) but haven't considered methods for the meta-analysis of rare events or how they handled studies with no events in one or more arms. Further details are provided in the Cochrane handbook https://training.cochrane.org/handbook/archive/v6.1/chapter-10#section-10-4-4.

Response: Thank you. We have amended our analyses of rare events. We analysed Hypoglycemia events using Mantel-Haenzel method, and Neurological Sequela events and ECG abnormalities with Peto's method. We have stated this in the Methods Section (see lines 155-158). Please note that the results compared to our initial analyses were similar.

7. In figure 1, 2 studies were excluded because the outcomes relevant to this review were not reported. Did the review authors attempt to contact the original study authors to obtain the necessary outcome data?

Response: Thank you. Two studies were excluded because they did not measure the outcomes of interest, not because they measured them but did not report them. We realized we may not have made this clear enough and we have therefore clarified this in S2 and Fig 1. Byakika-Kibwika et al only measured days to parasite clearance, and Barnes et al recorded proportion of patients with asexual parasitaemia of less than 60% after 12 hours.

8. In the results section the authors state: "A traditional meta-analysis conducted as

shown in S11, found that artemether…" but details of this analysis are not provided in the methods section.

Response: Thank you. We have amended this in the Methods Section (see lines 156-159).

9. Methods used to estimate the between-study heterogeneity and calculation of the 95% CIs needs to be explicitly stated in the methods section of the review.

Response: Thank you. We have amended this in the Methods Section (see lines 168-169).

10. Subgroup and sensitivity analyses need to be explicitly stated in the statistical methods section.

Response: Thank you. We have made this clearer by providing an Additional analyses subsection (see lines 178-183).

11. Throughout the manuscript, the authors rely on 'statistical significance' to draw their conclusions. The dichotomization of results based on statistical significance using a cut-off value of p<0.05 is strongly discouraged – see for example, one of amy articles on this topic, the paper by Greenland et al 2016. Further, there is no need to draw attention to certain values in your tables by bolding these values based on p-values <0.05.

Greenland, Senn, Rothman, et al. Statistical Tests, p-values, confidence intervals, and power: a guide to mininterpretations. Eur J Epi. 2016; 31(4): 337-350.

Response: Thank you and apologies for previously putting too much emphasis on statistical significance. Due to the large number of outcomes and drugs being compared, we thought it was useful to highlight significant results in the tables. We have now amended the manuscript throughout to lay less emphasis on statistically significant results. (see lines 257-264,308-310, 367-369, etc)

Minor edits:
1. line 68 – space between "mostdeadly" (i.e. most deadly)

Response: Thank you. This has been corrected.

2. Reference in line 123 should be reference 23 not reference 22.

Response: Thank you. This has been corrected.

3. Table 1:
o Months are written inconsistently. For example, October is sometimes written in full, other times as ‘Oct’ and also as ‘Oc’. Please write all months consistently throughout the table.

Response: Thank you. This has been corrected.

o Follow up days for study by Ojuawo et al 1998 is missing, so are the age (years) for this study.
Response: Thank you. This has been corrected.

o I would suggest using abbreviation ‘N/R’ (Not Reported) for information that wasn’t reported instead of the dash (e.g. when age wasn’t reported). This also applies when standard deviation or range was not reported for age. – use N/R instead of just reporting only the mean.
Response: Thank you. This has been amended as suggested.

o Please differentiate reporting of range and interquartile range for age. Range and inter-quartile range are different and should be differentiated in the table.

Response: Thank you. This has been clarified.

4. Table 2: Footnote “* Does not add up to 100% since two studies included both age groups and was omitted” should say “Does not add up to 100% since two studies included both age groups and were omitted”

Response: Thank you. This has been corrected.

5. The text in lines 161-163 does not directly reflect what is in Table 2. For example, the text says “All 17 RCTs from Africa were conducted among children” but in Table 2 only 16 studies conducted in Africa were in children.

Response: Thank you. We have amended this (see lines 208-210) to read “*Sixteen out of 17 RCTs from Africa were conducted among children and 11 out of 12 RCTs conducted among adults were in Asia. Out of the 12 cerebral malaria only RCTs, eight were from Africa and four from Asia.*”

6. Table S3 – the n’s in the first row don’t add up to the total. Same comment for row

with data for Africa and Asia (It looks like the numbers in the rows corresponding to Africa and Asia have been swapped).

Response: Thank you. S3 Table shows the frequency distribution of pairwise comparison across effect modifiers. The total number of pairwise comparisons are 41. We have cross checked data on S3 Table and they are all correct. To clarify we have added a footnote to this supplementary table.

7. Table 3 – it should be clear that Arteether has not been evaluated in adults, so the effect estimates can not be derived for adults (i.e. in the lower triangle of the figure).

Response: Thank you. This has been clarified in the results section (see lines 221 and 227) and the footnote of Table 3 (see line 268)

8. Please check statement in line 196 "…descending probability of being the best against quinine.”. The probabilities should be the probability of being the best. However, the effect estimates are compared to quinine – this sentence needs revising.

Response: Thank you. This has been amended (see line 230)

9. In the Discussion section, all effect estimates should be presented with their corresponding 95% confidence intervals.

Response: Thank you. We have amended this in the Discussion section (see lines 359-362).

Reviewer #2

This paper is a systematic review of all randomised trials in severe malaria comparing the artemisinin derivatives and/or quinine. The main novelty is the use of a network meta-analytic approach which allows for the use of indirect evidence to compare treatments for which there are few data from direct comparisons.

Overall I think the analysis is well done and the accompanying GitHub repository provides all the meta-data and the R scripts used to analyze the data. Everything is therefore easily reproducible and this provides a useful resource for future work.

My main issue is the how the results are summarized and how the evidence was assessed.

Major comments:

First of all, as in most research areas, there are many small randomised trials and only a few large definitive randomised trials. In this case there are only 4 large studies (>500 patients):
-Hien 1996 (n=560)
-Van Hensbroek 1996 (n=576)
-Dondorp 2005 (n=1461)
-Dondorp 2010 (n=5425)

These 4 studies (3 of which were run by the same research group with near identical designs and inclusion criteria) represent >70% of all randomised data. In addition Phu et al (2010, n=370) did the main head-to-head comparison of artesunate versus artemether. It is important to note that the Phu et al trial was done in the same patient population and the same hospital wards as Hien 1996 (in the years following Hien et al, but published much later), thus giving high internal validity to the artesunate vs artemether vs quinine 3 way comparison in adults.
What I can't understand is how the evidence from these studies, which dominate the available evidence base, can be called "moderate" and "low" quality evidence. As the authors know well, large studies are much less likely to suffer from publication bias, and have greater generalizability. The two largest studies (SEAQUAMAT and AQUAMAT) are both graded as fully "low risk" for the risk of bias (S4 Figure). However, Hien 1996 and Van Hensbroek 1996 both have "some concerns" for the randomization process, but this is very puzzling. Could this be explained? From the papers:
Hien 1996: "Patients were randomly assigned to receive artemether (50 mg per milliliter) or quinine dihydrochloride (250 mg per milliliter) for a minimum of 72 hours. The drugs were issued in packs of 10 identical 3-ml ampules by the Kunming Pharmaceutical Company (Kunming, People's Republic of China). [..]
The drugs for each patient were placed in a coded sealed envelope, and the envelopes were randomized in blocks of 20. Once a patient was enrolled in the study the envelope

was opened. Subsequent analysis of efficacy was on an intention-to-treat basis. Although the ampules, drug volumes, and administration schedules of artemether and quinine were identical, the viscosity and color of the two drugs were slightly different. To maintain blinding, a separate team of nurses, who were not otherwise involved with the care of the study patients, drew up and gave the injections. The drugs were kept in an opaque packet in a locked cabinet during the study."

Van Hensbroek 1996: "The treatment code for each child was stored in a sealed envelope that was opened after the admission procedure was completed and parental consent had been obtained. The different dose schedules used for artemether and quinine meant that the ward staff members were aware of the patients' treatment assignments. However, the assessment of neurologic sequelae in survivors was carried out by a doctor who was unaware of the treatment code."

In both cases the randomisation procedure seems more than adequate! Hien 1996 took special care to maintain blinding (more that what is usually done) and van Hensbroek 1996 was not blinded but the outcomes (other than mortality) were determined by a doctor blinded to assignment. In both cases, this is as close to gold standard procedures as is possible in low resource settings. I see very low risk of bias in both studies for the mortality endpoint.
Thus, when the abstract says "Compared to quinine, artesunate reduced mortality in children (risk ratio (RR), 0.76; 95%CI [0.65 to 0.89], moderate quality), adults (RR, 0.55; 95%CI [0.40 to 0.75], low quality) and in cerebral malaria (RR, 0.72; 95%CI [0.55 to 0.94], moderate quality)" I find this rather hard to reconcile with what i know about these trials!

Response: Thank you. The quality of evidence produced, was assessed using the Confidence in Network Meta-analysis (CINeMA) approach which classifies evidence as high, moderate, low or very low quality based on the within-study bias, heterogeneity, reporting bias, imprecision, indirectness and incoherence.

In CINeMA, the within-study-bias represents inadequacies in the conduct of research that can lead to deviation from the truth. This is measured using the assessment of risk of bias tool. The Reviewer raises important concerns which only relates to the within-study-bias. CINeMA considers these concerns by combining the studies' contributions with the risk of bias judgments to evaluate within-study bias for each estimate from a network meta-analysis. It uses the percentage contribution of each study and then computes the percentage contribution from studies judged to be at low, moderate, and high risk of bias. This is generated from the CINeMA webpage (https://cinema.ispm.unibe.ch/). Please, refer to this website and the documentation within that site for more details regarding the methodology.

We have adjusted our judgements on within-study-bias in the CINeMA webpage to accommodate the Reviewers concerns and have ammended this in S14. This is seen in the Contribution Matrix

presented below. However, we have maintained our judgement on Reporting Bias because both Egger's Test and Funnel plot indicates publication bias. Consequently, Artesunate vs Quinine for both age groups, are assigned Moderate Quality. We have amended S14 to capture details of the grading done with CINeMA.

Risk of Bias for Each Comparison in the Adult analyses

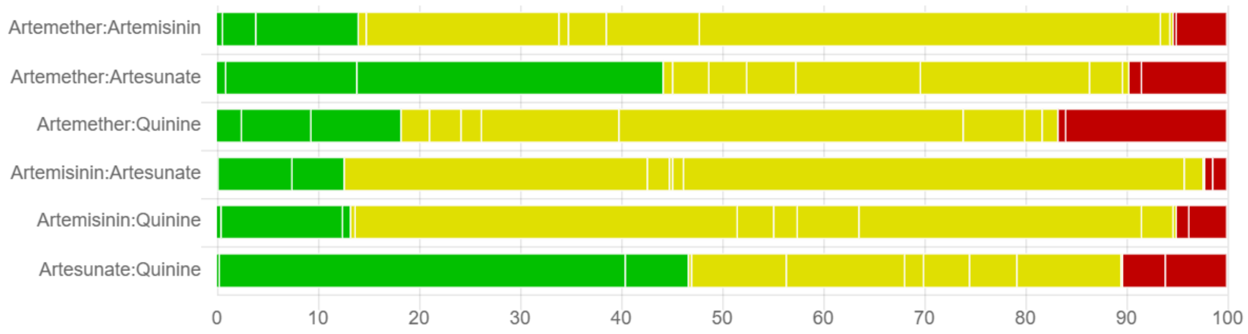

Risk of Bias for Each Comparison in the Children Analyses

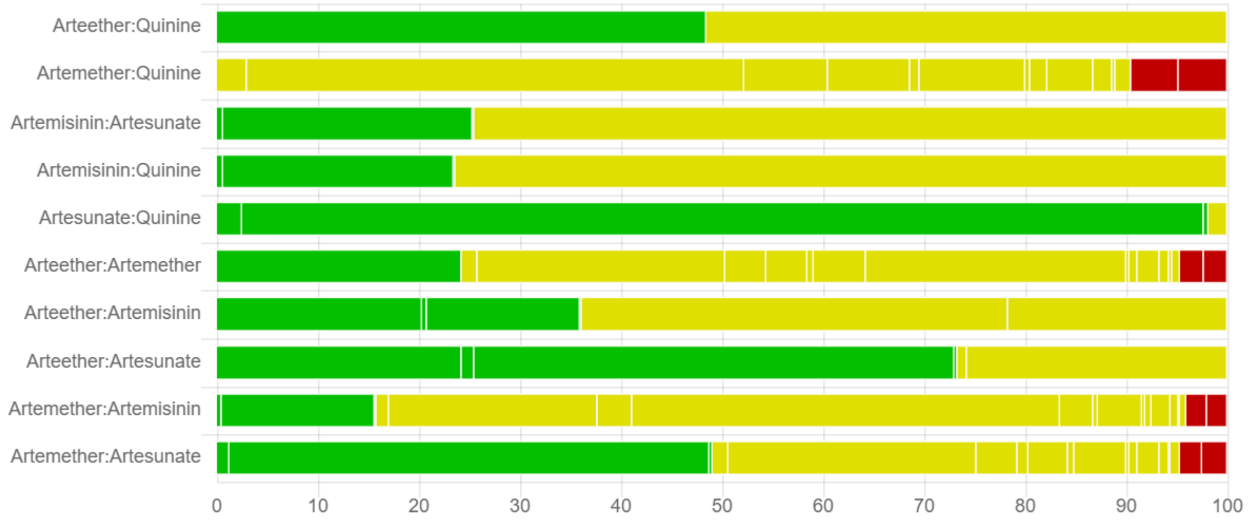

These figures represent the proportion of low, moderate or high risk of bias studies contributing to each comparison.

My second major comment concerns the fact that the analysis does not manage to establish a "batting order" for the three main drugs: artesunate, artemether and quinine. It's a near certainty that artesunate >> quinine (randomised trial evidence, biological mechanisms etc). It seems pretty clear that artesunate >> artemether (randomised evidence + pharmacokinetics). Although we expect treatment effects to differ between children and adults, we don't expect the rankings to change. So why not do a full network analysis including randomised trials in children and adults for artesunate,

artemether and quinine to determine the relative benefits of each? Again this would be primarily determined by Hien 1996, Hensbroek 1996, Dondorp 2005&2010 and Phu 2010…

Response: Thank you. We conducted sensitivity analyses lumping all studies regardless of age groups, as we indicated in our protocol. The results were similar. However, we did not add this to the Manuscript because we wanted to cut down on the word count. We have now amended the manuscript by mentioning this in the methods section (see line 183) and result section (lines 343-345) and provided the full results in S15 Table.

Response: Thank you. As for the ranking of treatments, we considered the results from this review including all randomized clinical trials (regardless of their sample size) comparing these drugs, and considering several outcomes, the evidence does not support that either artemisinin derivatives is superior to the others for all outcomes consistently. Therefore, the ranking of treatments was not consistent across outcomes. This is one of the main findings of this review. In this review, we did not systematically look at pharmacokinetics or studies detailing biological mechanisms, instead we looked at randomized controlled trials, which provide the best evidence for determining treatments effectiveness.

Minor comments:

Hypoglycaemia was not stated as an outcome in the earlier section of the Methods but then stated as an outcome later on.

Response: Thank you. We used adverse events to capture Hypoglycaemia and ECG abnormalities. We have amended this in the method by stating these adverse events (see lines 119-122).

Coma recovery time and other “intermediate” endpoints: estimates will be biased as you need to adjust for mortality as a competing risk. This can only be done with individual patient data.

Response: Thank you. We acknowledge the Reviewers concern that there is a more appropriate method for analyzing Coma Recovery Time using Individual Patient Data. However, that kind of data was not available so we used methods also used by previous Cochrane Systematic Reviews.

Fig 2: surely this makes no sense to show thickness in terms of the number of studies? The number of patients in all randomised comparisons is much more important (eg ten studies each with 50 patients, n total is 500, versus 1 study with 5000 patients!)
This is exactly what has happened with artesunate.

Response: Thank you. We acknowledge the Reviewers concern that it is important to represent the number of patients. As shown in the Figure 2 and legend, the blue nodes are proportional to the number of participants allocated to that drug. The thickness of the black edges is proportional to the number of studies comparing the drugs on each side of the edge.

Table 1 has some problems.
Hien 1996: the dates are stated in the paper ("Between May 1991 and January 1996, 561 patients were enrolled in the study")
Van Hensbroek 1996: not IV QN but intramuscular (abstract: We conducted a randomized, unblinded comparison of intramuscular artemether and intramuscular quinine in 576 Gambian children with cerebral malaria)

Response: Thank you. We have amended this in Table 1.

---

6. PLOS authors have the option to publish the peer review history of their article (what does this mean?). If published, this will include your full peer review and any attached files.

If you choose "no", your identity will remain anonymous but your review may still be made public.

Do you want your identity to be public for this peer review? For information about this choice, including consent withdrawal, please see our Privacy Policy.

Reviewer #1: No

Reviewer #2: **Yes:** James Watson

[NOTE: If reviewer comments were submitted as an attachment file, they will be attached to this email and accessible via the submission site. Please log into your account, locate the manuscript record, and check for the action link "View Attachments". If this link does not appear, there are no attachment files.]

While revising your submission, please upload your figure files to the Preflight Analysis and Conversion Engine (PACE) digital diagnostic tool, https://pacev2.apexcovantage.com/. PACE helps ensure that figures meet PLOS requirements. To use PACE, you must first register as a user. Registration is free. Then, login and navigate to the UPLOAD tab, where you will find detailed instructions on how to use the tool. If you encounter any issues or have any questions when using PACE,

please email PLOS at figures@plos.org. Please note that Supporting Information files do not need this step.
