# Supplementary material for: Comparative efficacy and safety of the artemisinin derivatives compared to quinine for treating severe malaria in children and adults: A systematic update of literature and network meta-analysis
Source: PLoS One. 2022 Jul 20;17(7):e0269391. doi: 10.1371/journal.pone.0269391 (PMC9299386; doi:10.1371/journal.pone.0269391)
Supplement: Attachment — Submitted filename: Response to reviewers.docx [file pone.0269391.s004.docx]

**REBUTTAL LETTER**

We thank the two reviewers for their comments on our manuscript and suggestions for improvement, which have been thoroughly incorporated into this revised version of our manuscript. Below is our response to each point raised by the reviewers. We hope that we satisfyingly addressed them and that the manuscript will be now suited for publication.

Sincerely,

On behalf of all authors

Nicholas Nyaaba (RGN, MSc)

**Reviewer #1**

Reviewer #1: Further comments specific to the current manuscript and the authors response to the reviewers’ comments are provided below:
1. Transitivity requires that the requirements below are satisfied:
a. The treatments included in the meta-analysis are considered to be ‘jointly randomizable’,

b. The different trials are similar enough to be combined,

Response: Thank you. We had considered this assumption but had not included details in order to keep within the word count. We have now added an ‘Interventions’ section and more details to the “Inclusion and Exclusion Criteria” section to show that the treatments and participants were ‘jointly randomizable’. (Line 105-116, Line 125-128, Line 155-157)

*“Interventions*

*This review covered parenteral and rectal artemisinin drugs administered during the critical phase of treating P. falciparum severe malaria until oral antimalarial can be tolerated by the patient. Parenteral interventions cover both IV and IM interventions. Just as IV and IM artesunate are considered to be similar^5^, IV and IM quinine do not have significantly different therapeutic benefits in treating severe malaria.^65,66^ Rectal artemisinin has been compared to parenteral quinine and artesunate in previous studies and has been found to be equally effective in treating severe malaria.^48–50^ Therefore, both parenteral and rectal interventions were pooled together in the analyses. However adverse events were analyzed and interpreted with the assumption that local reactions will differ with route of administration. Different dosages for the interventions within recommended ranges were combined into a single node, therefore, trials comparing the same drug at different doses were excluded.”*

*“The criteria for inclusion ensured that all RCTs included in this review compared the artemisinin derivatives and quinine for the treatment of P. falciparum among adults and children, in head to head comparisons. Children were considered as those aged < 15 years. Trials which included pregnant women were excluded.”*

*“The interventions in the study were similar in all comparisons. Also, the participants in both adult and children analyses were similar in all studies and could have been assigned to any of the treatments as indicated above, therefore, meeting the joint randomisability requirement for NMA.”*

c. The characteristics associated with the effect of the treatments are similar across the included trials (Salanti 2012 and Chaimani et al 2021).
Requirements a and b need to considered at the planning stage of the network meta-analysis, and requirement c is assessed after the data from each of the included trials has been collected. The authors have only assessed requirement c and have not considered requirements a and b.

In addition, in response to the other reviewer, the authors have presented the results all studies (i.e., combining trials in adults and children together). However, this again, is only justifiable if the transitivity assumption is adhered to (see requirements a above). If the treatments given to children and to adults cannot be considered to be jointly randomizable then it does not make sense to present these results pooled together.

Response: Thank you. We acknowledge the Reviewer’s concern so we took steps to address this in our analyses. Separate analyses were conducted for adults and children to reduce clinical inconsistency and heterogeneity, for the main analyses. However, we only pooled all studies regardless of age groups in sensitivity analyses. We have added a sentence to the “Assessment of Transitivity” section to clarify this. (Line 155-157, Line 169-170)

*“The interventions in the study were similar in all comparisons. Also, the participants in both adult and children analyses were similar in all studies and could have been assigned to any of the treatments as indicated above, therefore, meeting the joint randomisability requirement for NMA.”*

*“Separate analyses were conducted for adults and children to reduce clinical inconsistency and heterogeneity, as well as meet the transitivity requirement”*

2. The authors have claimed that they followed the methods recommended in the Cochrane Handbook for analysis of skewed data. However, the Cochrane handbook states that “analyses based on means are appropriate for data that are at least approximately normally distributed… Review authors should consider the possibility and implications of skewed data when analysing continuous outcomes” (section 10.5.3 <https://training.cochrane.org/handbook/current/chapter-10#section-10-5-3>). Further, they recommend that “Collection of appropriate data summaries from the trialists, or acquisition of individual patient data, is currently the approach of choice.” (section 10.5.3 <https://training.cochrane.org/handbook/current/chapter-10#section-10-5-3>).

Response: Thank you. We acknowledge the Reviewer’s concern. Based on the recommendations of the Reviewer and the Cochrane Handbook for Systematic Reviews we have compared continuous outcomes using mean difference for the main analyses and have only added studies in which we made conversions in the sensitivity analyses. (Line 147-149, Table 3, 300-335)

*“Where medians and range or interquartile range were reported instead of the means and standard deviations, the latter were estimated using Wang’s method [28]. These estimations were only used in sensitivity analyses*.”

3. I agree with the other reviewer that showing the thickness of the edges in the network map as number of studies doesn’t make sense. I would strongly suggest that the authors consider changing this to correspond to the precision of the estimate instead of the number of studies.

Response: Thank you. We acknowledge the Reviewer’s concern that it is important to represent the number of patients. As shown in the Methods (Line 182-184) nad Figure 2 legend (Line 266-268), the blue nodes are proportional to the number of participants allocated to that drug. The thickness of the black edges is proportional to the number of studies comparing the drugs on each side of the edge. We have followed the convention for network graphs which addresses the Reviewers’ concern, and illustrates both precision (with the size the nodes) and then the number of studies with the edges.

*“The bigger the size of the nodes, the greater the pooled sample size in the treatment. The thicker the edges, the greater the number of studies comparing the treatment.”*

*“The blue nodes are proportional to the number of participants allocated to that drug. The thickness of the black edges is proportional to the number of studies comparing the drugs on each side of the edge.”*

*Chaimani A, Higgins JPT, Mavridis D, Spyridonos P, Salanti G (2013) Graphical Tools for Network Meta-Analysis in STATA. PLoS ONE 8(10): e76654.* [*https://doi.org/10.1371/journal.pone.0076654*](https://doi.org/10.1371/journal.pone.0076654)*.* [*https://journals.plos.org/plosone/article?id=10.1371/journal.pone.0076654*](https://journals.plos.org/plosone/article?id=10.1371/journal.pone.0076654)

4. The authors should make it clear which analyses were added post hoc.

Response: Thank you. We confirm that all the analyses were pre-specified as detailed in the protocol.

*Nyaaba N, Ordonez-Mena J, Hirst J. What are the comparative efficacy and safety of the artemisinin derivatives for treating severe malaria? [Internet]. PROSPERO- International prospective register of systematic reviews; 2020.*

[*https://www.crd.york.ac.uk/prospero/display_record.php?ID=CRD42020218190*](https://www.crd.york.ac.uk/prospero/display_record.php?ID=CRD42020218190)

5. The link to the GitHub code does not work. I received a ‘404’ error message when I tried to access it.

Response: Thank you. Apologies. This error has been fixed. [Github Account](https://github.com/nyaaba-nick/A-Comparative-Efficacy-and-Safety-of-the-Artemisinin-Derivatives-a-Network-Meta-Analyses-R-Script-.git)

6. The netheat plot and forest plot of direct and indirect estimates are not presented for the primary outcome (mortality). Please explain or provide.

Response: Thank you. We did not provide netheat plots and forest plots for mortality because the Q and I^2^ statistics did not indicate any evidence of substantial variability in the effect sizes for mortality. We only used the netheat plots, forest plots of the direct and indirect evidence, and net splitting methods when there was substantial evidence of inconsistency in the network for a particular outcome. We have now made this clear in the Methods section. (Line 193-195)

*“Analyses which recorded substantial inconsistencies were further investigated to identify hotspots using net-splitting and design by treatment methods as appropriate, and illustrated with the net-splitting and net heat plots respectively [36,37].”*

7. The estimates reported in the manuscript in lines 322-325 are RRs but are referencing the figure S11, which shows odds ratios. This is very confusing. This applies to the estimates in lines 327-330 and Figure S12.

Response: Thank you. We converted the ORs from S9 and S10 B into RRs for easy interpretation and consistency. We have now reported both ORs and RRs for these results as indicated in the Methods (Lines 178-179) and Result sections (Lines 338-342, 350-353).

*“ We however interpreted these results using RR by calculation from the Odds Ratios (OR) using the assumed comparator risk which was arbitrarily chosen as the median comparator risk [19].”*

*“**We have converted ORs in S9 and S10 B to RRs for easy interpretation but the overall direction on the forest plots remain the same. Traditional meta-analysis pooling 10 RCTs showed that artemether (OR, 0·87; 95%CI [0·55 to 1·37], RR, 0·87; 95%CI [0·56 to 1·34]) may reduce neurological sequela events compared to quinine with mild heterogeneity (I^2^=28%, p=0·22). Results can be seen in S9 Fig.*

*“A traditional meta-analysis conducted as shown in S10 B, found that artemether (OR, 1·72; 95%CI [0·96 to 3·05], RR, 1·65; 95%CI [0·96 to 2·75]) may increase the number of electrocardiogram abnormalities compared to quinine, and this was associated with moderate heterogeneity (I2=69%, p=0·02).”*

8. In lines 322-325, it should be clear how many studies are contributing to the pooled estimates. Most of the comparisons in Figure S11 are from 1 study.

Response: Thank you. We have included this in Line 340.

*“Traditional meta-analysis pooling 10 RCTs showed that artemether (RR, 0·87; 95%CI [0·56 to 1.34]) may reduce neurological sequela events compared to quinine with mild heterogeneity (I^2^=28%, p=0·22). Results can be seen in S 9 Fig.”*

9. In the text, the authors state that ‘Figure S11’ is a network forest plot but examination of Figure S11 looks like a pairwise Forest plot. Please clarify. Further, if the estimates are from a network meta-analysis it needs to be clearer how Peto method was invoked in the netmeta package in R.

Response: Thank you. We have clarified this; S11( now S9) and S10 B are traditional Meta-analysis with Peto’s Method while S10 A is network meta-analysis with Mantel Haenszel method. (Line 338, 345)

10. For Figure 5, it needs to be clear what is being plotted here – is it just the estimates for artemisinin vs quinine (as reads in the manuscript lines 347/348)? If so, where are the forest plots for the remaining analyses?

Response: Thank you. The comparison adjusted funnel plot represents all the artemisinin drugs. We have clarified this in Line 369-370.

*“Smaller studies were more likely to produce a beneficial effect in favour of all the artemisinin drugs.”*

11. Minor comments:

a. There are some grammatical errors:
i. For example, lines 126/127 “For binary outcomes, the number of participants 127 experiencing the event and numbers assessed in each randomised group was recorded.” Should be “For binary outcomes, the number of participants 127 experiencing the event and numbers assessed in each randomised group were recorded.”
ii. Similarly, line 152 “Risk ratios was pooled… “ should be “Risk Ratios were pooled…”
iii. Netheat is sometimes spelled as two words ‘net heat’ and other times as one word ‘netheat’, please make it consistent throughout the manuscript (including in the figures).

Response: Thank you. These have been corrected. (Line 143,172). It has now been changed to “net heat” plots.

b. Lines 202-203, please add references consistently – i.e., you have provided the reference for the trial completed in South Pacific but have not provided the references for the trials completed in Asia or Africa. This applies to all references to trials in this paragraph and throughout the manuscript.

Response: Thank you. This has been corrected. We have provided a general reference to Table 1 in Line 225.

c. The age group for the study by Haroon et la (ref #39) in Table 1 is missing.

Response: Thank you. We apologise. This has been inserted.

d. Lines 205-206 state that “Twelve RCTS were among participants with only cerebral malaria…” but I count 13 in Table 1.

Response: Thank you. We have corrected this. Olumese et al was incorrectly entered as cerebral malaria.

e. In table 3, it needs to be clear in the footnote which test was used to generate the p-value for heterogeneity.

Response: Thank you. We have added a statement to indicate this. Line 297-298.

*“The DerSimonian and Laird method was used to estimate the between-study variance, and the Jacksons method to estimate its confidence intervals [35,31]*

*P-values were two-sided with a significance level of 0.05.”*

f. In Table 3, it needs to be clear in the footnote how inconsistency was assessed.

Response: Thank you. We have added a sentence to show this. Line (299)

“*Cochran Q method was used to assess the total variability and then decomposed into heterogeneity and inconsistency [36].”*

g. In Table 3, it needs to be clear what the difference is between total variability and heterogeneity, and how they are calculated.

Response: Thank you. We have added a sentence in the “Assessment of Heterogeneity and Inconsistency” section. (Line 190-192)

“*The variability among the individual study estimates as compared to the network estimates was represented by the total variability, using the Cochran Q method [32]. This was then broken down into within-design (heterogeneity) and between-design (inconsistency).*”

12. A minor comment to the authors for future response to reviewers – I find it helpful if the response indicates the specific changes that are made and corresponding line numbers. This makes it easier as a review to identify exactly how the authors have addressed the concerns raised.

Response: Thank you. We have added line numbers when responding to the reviewers’ comments.


References
Salanti G. Indirect and mixed-treatment comparison, network, or multiple-treatments meta-analysis: many names, many benefits, many concerns for the next generation evidence synthesis tool. Research Synthesis Methods 2012; 3: 80–97.
Chaimani A, Caldwell DM, Li T, Higgins JPT, Salanti G. Chapter 11: Undertaking network meta-analyses. In: Higgins JPT, Thomas J, Chandler J, Cumpston M, Li T, Page MJ, Welch VA (editors). Cochrane Handbook for Systematic Reviews of Interventions version 6.2 (updated February 2021). Cochrane, 2021. Available from [www.training.cochrane.org/handbook](http://www.training.cochrane.org/handbook).

Deeks JJ, Higgins JPT, Altman DG (editors). Chapter 10: Analysing data and undertaking meta-analyses. In: Higgins JPT, Thomas J, Chandler J, Cumpston M, Li T, Page MJ, Welch VA (editors). Cochrane Handbook for Systematic Reviews of Interventions version 6.3 (updated February 2022). Cochrane, 2022. Available from [www.training.cochrane.org/handbook](http://www.training.cochrane.org/handbook).

**REVIEWER #2**

Reviewer #2: Some final comments I would like to see addressed before publication:

Introduction, paragraph 2: please make it clear here that you are focusing on severe falciparum malaria. Falciparum and knowlesi are the two parasites which sequester in the microvasculature thus causing severe disease. Severe vivax is very different in terms of presentation and the exact definition is debated.
In addition severe falciparum is diagnosed from evidence of *asexual* stages in circulation.

Response: Thank you. We have added a statement to indicate this. (Line 65-68)

*“Malaria is febrile illness that is spread through the infected bites from female Anopheles mosquitos and it is caused by the Plasmodium spp (P.malariae, P.falciparum, P.vivax, P.knowlesi, P.ovale and P.cynomolgi), of which P.falciparum is the major cause of severe illness, therefore, severe P.falciparum malaria is the focus of this study [3].”*

Results: surely Figure 2 is wrong? In panel A the circle for ASU (artesunate) is very small, whereas ~3000 patients were randomised to ASU? Maybe I am missing something, but I don't quite understand this Figure.

Response: Thank you. Response: Thank you. Apologies. The weighting for the nodes was incorrect. This has been corrected. The size of the blue nodes are now proportional to the number of participants allocated to that drug.

Discussion: the authors write: “All artemisinin drugs except artemether may increase neurological sequela events in the first three to seven days of treatment, compared to quinine. Most of the neurological sequela events resolved during follow up, which is similar to findings of other studies [5,40,53].”
This should be caveated by the bias I mentioned in my first review: death and coma recovery are competing events: a drug that saves lives in patients who would have died with a different drug could thus result in longer coma recovery times (those patients would have died!). The authors do not have access to IPD and thus the effect estimates are likely to be biased.

Response: Thank you. We acknowledge that this is an important addition to the limitations of this study so we have included this in the Discussion. (Line 413, 425-427)

*“However, we observed that mortality is a competing event with these adverse outcomes.”*

*“Mortality may be considered as a competing event with some of the secondary outcomes but we were unable to explore this using more appropriate methods because individual participant data was not available.”*

In response to reviewer comments, the authors wrote:

“We have adjusted our judgements on within-study-bias in the CINeMA webpage to accommodate the Reviewers concerns and have ammended this in S14. This is seen in the Contribution Matrix presented below.”

However when I look at the Sup Material page 5, I still see a yellow "!" for Hien 1996 and Van Hensbroek 1996. Have the authors changed their assessment of “concerns about the randomization process” for these two studies? I strongly believe that the randomization process was more than adequate in these two studies as explained in detail in the first review.

Response: Thank you. We apologise for the omission. The initial change after the first review was made in the quality of evidence assessment, we have now effected this change on the Risk of Bias Chart (S4) and the manuscript (Line 244).

The authors wrote:
“This is one of the main findings of this review. In this review, we did not systematically look at pharmacokinetics or studies detailing biological mechanisms, instead we looked at randomized controlled trials, which provide the best evidence for determining treatments effectiveness.”

For death - the main outcome we care about in the treatment of severe malaria (a disease with 10-30% mortality!) - it is pretty clear that artesunate is better than quinine. I agree that RCTs are the best evidence here.
For parasite clearance, this is also very clear: this is the main surrogate marker of drug activity in vivo.
Neurological sequelae have not be very well documented in these trials as it is very hard to consistently follow patients up after hospital discharge (Hien 1996 is only large trial to have done proper follow-up). As I have stated before, coma recovery times based on aggregated data are biased. So, no, I do not think that a main finding is a lack of consistency. A main finding is lack of data or lack of access to IPD for these outcomes. IPD meta-analysis would be much better to estimate the effects for these secondary outcomes.

Response: Thank you. We acknowledge that this is an important addition to the limitations of this study so we have added a statement in the Discussion to reflect this. (Line 425-427)

*“Mortality may be considered as a competing event with some of the secondary outcomes but we were unable to explore this using more appropriate methods because individual participant data was not available.”*
